# Biological Function of Medium-Chain Fatty Acids and Their Application in Aquatic Animals: A Review

**DOI:** 10.3390/ani15152294

**Published:** 2025-08-06

**Authors:** Haiyan Liu, Wenzong Zhou, Chenggang Cai, Fengqin Feng, Haiying Cai, Hang Yang

**Affiliations:** 1 Eco-Environmental Protection Research Institute, Shanghai Academy of Agricultural Sciences, Shanghai 201403, China; 18883681960@163.com (H.L.); zhouwz001@126.com (W.Z.); 2 Key Laboratory of Integrated Rice-Fish Farming Ecosystem, Ministry of Agriculture and Rural Affairs, Shanghai Academy of Agricultural Sciences, Shanghai 201403, China; 3 College of Biology and Chemical Engineering, Zhejiang University of Science and Technology, Hangzhou 310023, China; ccg516@sina.com; 4 School of Biosystem Engineering and Food Science, Zhejiang University, Hangzhou 310058, China; feng_fengqin@hotmail.com

**Keywords:** aquatic animals, growth performance, immunity, intestinal flora, medium-chain fatty acid triglycerides (MCTs)

## Abstract

Medium-chain fatty acid triglycerides (MCTs) possess antibacterial, antiviral, nutritional, and other biological activities and have demonstrated significant application potential in humans and terrestrial animals. In recent years, with the development of the green aquaculture industry, MCTs have been gradually applied to aquaculture animals, which can enhance growth performance, improve flesh quality, regulate lipid metabolism, boost immune activity, and modulate the intestinal flora, thereby improving the production efficiency of aquaculture. This paper elaborates in detail on the biological activities of MCTs and their applications in aquatic animals, providing a theoretical and practical basis for the application of MCTs in aquaculture.

## Simple Summary

Medium-chain fatty acid triglycerides offer multiple benefits for aquatic animals and sustainable aquaculture. This review explores how medium-chain fatty acid triglycerides improve fish and shrimp health by enhancing growth, boosting immunity, and optimizing gut bacteria. Studies show that medium-chain fatty acid triglycerides reduce disease outbreaks, improve meat quality (e.g., higher healthy fats and better texture), and help fish digest nutrients more efficiently. By replacing conventional feed additives, medium-chain fatty acid triglycerides support eco-friendly aquaculture practices, ensuring safer seafood production, and reducing the reliance on antibiotics. These findings provide farmers with science-backed strategies to raise healthier aquatic animals, benefiting both the industry and consumers. Future research will focus on tailoring medium-chain fatty acid triglycerides use for different species and environments to maximize their potential.

## 1. Biological Activities of Medium-Chain Fatty Acid Triglycerides

In recent years, the global aquaculture industry has been facing multiple challenges, which stem from both the inherent contradictions of industrial-scale development and the external drivers of ecological security and consumption upgrading. First, intensive high-density farming models (such as shrimp high-position ponds [1] and cage fish farming [2]) have met protein demands by maximizing the production capacity per unit water body [3], but they have also completely altered the living environment of aquatic organisms [4], leading to an increased risk of disease outbreaks (such as Vibrio infection [5] and hepatopancreatic necrosis [6]). Second, the global protein consumption structure is undergoing profound changes: the United Nations Food and Agriculture Organization (FAO) predicts that per capita aquatic product consumption will increase from 20.2 kg in 2020 to 21.4 kg in 2030, a 15% growth [7], and consumers’ demands for aquatic food are gradually shifting from “quantitative safety” to “quality safety”. Finally, the safety controversies of feed additives remain a prominent challenge in the current farming environment. In the current aquaculture feed additive market, the use of antibiotic growth promoters and chemical preservatives still dominates, but their side effects are gradually emerging [8]. The abuse of traditional antibiotics can trigger a chain reaction of drug resistance, food safety, and ecological toxicity [9,10]. Therefore, the research and development (R&D) and application of novel feed additives have become a key breakthrough to address the industrial dilemmas.

**Figure 1:**
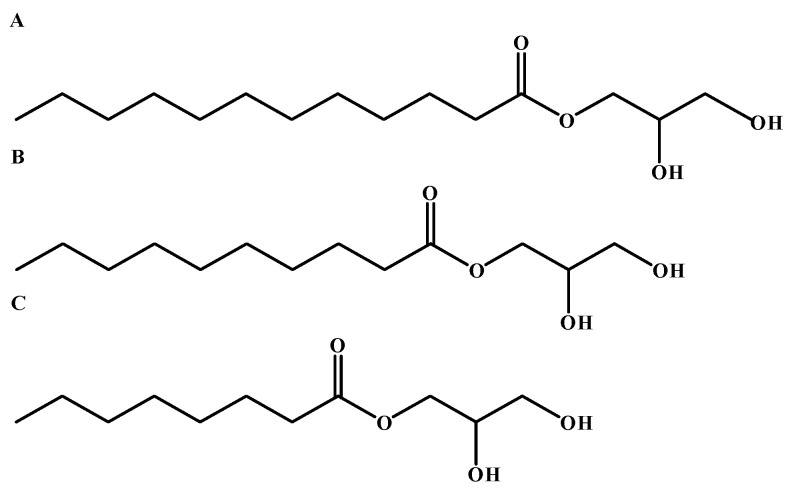
Chemical structure of monoglycerides of medium-chain fatty acid, (**A**) monoglycerides of lauric acid; (**B**) monoglycerides of capric acid; (**C**) monoglycerides of caprylic acid.

Medium-chain fatty acid triglycerides (MCTs) are esters composed of three medium-chain fatty acids (MCFAs, 6–12 carbon atoms) esterified to a glycerol backbone, distinct from monoglycerides and diglycerides [11]. Representative monoglycerides include glycerol monolaurate (GML), glycerol monodecanoate (GMD), and glycerol monocaprylate (GMC), with their structural formulas illustrated in Figure 1. Common triglycerides comprise glycerol caprylate (C_27_H_50_O_6_), capric acid triglycerides (C_33_H_62_O_6_), and mixed caprylic-capric acid esters [12]. The primary natural sources of MCTs include milk fat, coconut oil, and palm kernel oil. Coconut oil contains 45–52% lauric acid (C12:0), 5–10% caprylic acid (C8:0), and 4–8% capric acid (C10:0) [13], while palm kernel oil consists of 2.4–6.2% caprylic acid, 2.6–7.0% capric acid, and 41–55% lauric acid [14]. In comparison, milk fat has a lower MCT content, accounting for only 4–12% of total fatty acids [15]. At room temperature, MCTs are colorless, non-irritating oily liquids characterized by low molecular weight and short carbon chains. They are insoluble in water but readily soluble in organic solvents such as ethanol. Due to the properties of their constituent MCFAs, including low melting/boiling points, small molecular size, and low density, MCTs demonstrate superior oxidative stability compared to conventional fats and hydrogenated oils, attributed to their minimal unsaturated fatty acid content (iodine value < 0.5). Furthermore, their low surface tension, high ductility, and excellent solubility allow them to dissolve various compounds, including vitamins, biocides, and hormones, making them valuable in pharmaceutical, nutraceutical, and animal feed applications. The short carbon chain structure of MCTs prevents gastric hydrolysis, enabling direct entry into the small intestine. There, pancreatic lipase efficiently hydrolyzes them into MCFAs and glycerol. Notably, while MCTs exhibit higher water solubility than long-chain triglycerides (LCTs), a degree of emulsification remains necessary for effective digestion, albeit with reduced dependency on bile salt emulsification compared to LCTs [16]. The liberated MCFAs are absorbed by enterocytes via the intestinal villi’s microvilli and transported directly to the liver through the portal vein for rapid β-oxidation and energy production. In contrast, long-chain triglycerides (LCTs) must be re-esterified into triglycerides within enterocytes, incorporated into chylomicrons, and delivered to the liver via the lymphatic system before storage or energy conversion [17]. Consequently, MCTs exhibit significantly higher absorption efficiency than LCTs, provide rapid energy, and remain well-absorbed even under pancreatic insufficiency. Therefore, in response to the specific needs of aquaculture (disease prevention, sustainable development, and nutritional health), MCTs align with industry requirements due to their unique metabolic properties. They can replace partial antibiotic growth promoters, offering new approaches to address nutritional and health issues in farmed animals and facilitating the industry’s transition toward green and sustainable development.

### 1.1. Antimicrobial Activity

Domestic and international scholars have delved into the antimicrobial effects and mechanisms of MCTs, with GML showcasing remarkable antibacterial performance. GML’s antimicrobial activity surpasses that of free fatty acids, characterized by a broad spectrum, high stability, and negligible pH influence. GML exerts inhibitory effects on diverse microorganisms, displaying selectivity, which powerfully inhibits Gram-positive bacteria (e.g., *Staphylococcus aureus*), followed by specific Gram-negative bacteria, though its effect on *Escherichia coli* and *Salmonella* is relatively weaker [18,19]. Additionally, GML can inhibit filamentous fungi, yeasts, certain viruses, and protozoa [20]. Numerous studies have affirmed GML’s significant inhibition of bacterial spores. Kimsey et al. [21] discovered that GML can substantially decrease spore heat resistance, doubling or tripling the spore inactivation rates at 113–121 °C. At concentrations between 0.4 and 3.6 mmol/L, GML both prevented spore germination and directly eliminated spores. GML’s minimum inhibitory concentration (MIC) against *Bacillus subtilis*, *Bacillus cereus*, and *Staphylococcus aureus* could reach as low as 0.017 mg/mL in Feng’s study [22]. Schlievert et al. showed that GML at concentrations over 10 μg/mL can notably inhibit *Bacillus anthracis* growth, and concentrations of 5% (50,000 μg/mL) or more could effectively kill *Bacillus* and *Clostridium spores* [23]. Zhang et al. found that triglycerol monolaurate (TGML)’s MIC and minimum bactericidal concentration (MBC) against common foodborne pathogens were lower than those of traditional preservatives such as sodium benzoate and potassium sorbate. Its antimicrobial mechanisms mainly involve disrupting cell membrane integrity, enhancing membrane permeability, and impeding biomacromolecule synthesis [24]. Yoon et al. [25] revealed that GML’s antibacterial activity against *S*. *aureus* is 200 times stronger than that of lauric acid. Umerska et al. [26] developed GML-incorporated lipid nanocapsules (LNCs) with superior antimicrobial activity compared to fatty acid-LNCs. Notably, GML-LNCs were active at lower concentrations and cause hemolysis only at higher concentrations, hinting at their potential as antimicrobial drug carriers. Adding GML (8 mg/disc) to infant formula could significantly suppress the growth of various pathogenic bacteria, and 5 mg/disc suffices to effectively inhibit methicillin-resistant *Staphylococcus aureus* (MRSA) and Listeria monocytogenes [27]. As summarized in Table 1 for MCT additives, with its unique advantages, GML, as an efficient and safe antimicrobial agent, holds broad application prospects in food preservation, pharmaceuticals, and other fields.

MCTs inhibit bacteria via the following: (1) disrupting cell walls/membranes, causing leakage; (2) increasing membrane permeability, damaging DNA; (3) disrupting energy production.

GML is absorbed via the lymphatic system, enhancing its antimicrobial effects before liver degradation.

### 1.2. Antiviral Effects

GML stands out among MCTs for its significant antiviral properties, while other MCTs remain poorly characterized in this regard. It reduces viral infectivity by disrupting viral envelopes and causing content leakage. When combined with fatty acids, GML’s antiviral effect is enhanced, particularly against the herpes simplex virus through interactions with its lipid bilayer [28]. GML has demonstrated broad-spectrum activity against viruses including RSV, HSV-1, and influenza viruses through both direct mechanisms (membrane disruption, replication inhibition) and indirect mechanisms, effectively blocking the infection cycles of influenza, stomatitis, and polioviruses [29]. In gel formulation, GML destabilized viral membranes and inhibited infection-related signaling molecules, showing preventive efficacy against HIV in macaque models [30]. It also provided protection against vaginal viral transmission through combined antiviral and immunomodulatory effects, as demonstrated in SIV-macaque studies where pre-exposure GML administration prevented infection despite repeated high-dose challenges [31]. Additionally, tampons containing 1.4% GML reduced toxic shock syndrome incidence by inhibiting toxin production in *Staphylococcus* and *Streptococcus* species. This likely occurs through membrane-mediated interference with toxin gene expression, potentially via the agr regulatory pathway or modulation of environmental factor responses [32]. In summary, GML primarily disrupts the membrane structure of viruses through several mechanisms: (1) impairing their infectious ability; (2) stimulating the immune response and activating immune cells to enhance the defense against viral infections; and (3) inhibiting the production and transmission of signaling molecules associated with viral infection to reduce the efficiency and extent of viral spread.

### 1.3. Nutritional Effects

#### 1.3.1. Improvement of Lipid Metabolism

During lipid digestion, pancreatic lipase exhibits sequentially decreasing hydrolytic activity toward triglycerides, diglycerides, and monoglycerides (1-monoglycerides and 2-monoglycerides), with the weakest activity observed for 2-monoglycerides. MCTs can directly enter the liver via the portal vein, where they are rapidly metabolized for energy, thereby reducing fat accumulation [33]. A portion of MCTs is absorbed intact by intestinal epithelial cells entering the lymphatic system and bloodstream, where they exert antimicrobial and antiviral effects [34]. The remaining fraction is hydrolyzed by glycerol fatty acid enzymes into free fatty acids for absorption [35]. GML may influence lipid metabolism by stimulating the secretion of cholecystokinin (CCK), a gastrointestinal peptide hormone that enhances the activity of digestive enzymes for lipids and proteins, thereby modulating lipid metabolism [36]. Studies have shown that 0.15 g/kg of GML upregulates the expression of PPARα and ACOX1 in the liver, promoting fatty acid oxidation and energy expenditure while reducing triglyceride (TG) levels and fat storage. In contrast, 1.6 g/kg of GML increases the expression of PPARγ2 and CD36, potentially inhibiting fat accumulation and promoting thermogenesis in brown adipose tissue [37]. However, other research indicated that 0.15 g/kg of GML may elevate serum levels of TG, low-density lipoprotein (LDL), and LPS, while decreasing high-density lipoprotein (HDL), increasing the body fat percentage and epididymal adipose mass. These findings suggest that low-dose GML promotes fat deposition in mice, raising concerns about its long-term metabolic impacts [38]. In comparison, 1.6 g/kg of GML significantly reduced the epididymal adipocyte size in high-fat diet (HFD)-fed mice, suppressing fat accumulation. Additionally, GML elevated serum adiponectin levels while reducing leptin and hepatic TNF-α (a pro-inflammatory cytokine) expression, thereby mitigating inflammation [39]. Furthermore, GML decreased the abundance of obesity-associated genera such as *Bacteroides*, *Dorea*, and *Eggerthella*, improving the gut microbiota composition. Further mechanistic studies suggested that GML ameliorates HFD-induced lipid metabolism disorders by modulating the gut microbiome, thereby reducing the obesity risk [40]. Bile acids play a crucial role in lipid metabolism, and GML may also improve lipid metabolism by regulating bile acid levels [41], and GML may also improve lipid metabolism by regulating bile acid levels [42]. Additionally, compared to olive oil, MCT oil significantly reduces body weight, fat mass, and visceral fat, and increases energy expenditure and fat oxidation rates in overweight individuals, indicating its potential for fat loss [43,44]. The dose-dependent paradoxical effects of GML on lipid metabolism fundamentally derive from the dynamic equilibrium between pro-inflammatory and metabolic protective mechanisms. At low concentrations, GML induces the hepatic upregulation of PPARα and ACOX1 to enhance fatty acid β-oxidation, while concomitantly elevating serum TG, LDL, and lipopolysaccharide (LPS) levels. This dual effect promotes adipose deposition through two interrelated pathways: (1) LPS-mediated metabolic disorders via TLR4/NF-κB inflammatory signaling, and (2) reduction in the abundance of beneficial bacteria such as *Bacteroides*, disruption of intestinal barrier function, increased endotoxin translocation into the bloodstream, and the indirect promotion of adipose tissue inflammation and lipid accumulation. In contrast, high-dose GML shifts the regulatory axis toward metabolic homeostasis by activating brown adipose tissue thermogenesis (via the PPARγ2/CD36 pathway), inhibiting pro-inflammatory cytokines (TNF-α, leptin), and regulating enterohepatic bile acid circulation.

In conclusion, GML modulates lipid metabolism through multiple pathways, but its effects may vary depending on the dosage and metabolic context.

#### 1.3.2. Improvement of Intestinal Microecology

The *Firmicutes*/*Bacteroidetes* (F/B) ratio, typically 10.9:1 in healthy adults [45], is elevated in obesity. Gut microbiota dysbiosis contributes to metabolic disorders, with HFD promoting LPS-producing bacteria and systemic inflammation [46]. GML demonstrates significant microbiota-modulating effects, reducing *Firmicutes* and *Proteobacteria* while alleviating obesity-related inflammation [47]. In DSS-induced colitis mice, 0.5 g/kg of GML administration downregulated pro-inflammatory cytokines (TNF-α, IL-1β) and upregulated anti-inflammatory cytokines (IL-10, TGF-β), while enhancing intestinal barrier function through tight junction protein expression [48]. Sinisa et al. [49] demonstrated that coconut oil could increase the abundance of probiotics such as *Lactobacillus*, *Allobaculum*, and *Bifidobacterium* in rat feces, thereby altering the gut microbiota composition (with an increase in *Bacteroidetes* and *Actinobacteria*, and a decrease in *Spirochaetes*). GML supplementation increases microbial α-diversity and beneficially alters β-diversity, with specific correlations observed between microbial composition and metabolic markers [37]. Notably, *Lactobacillus* abundance correlated with lipid profiles, while *Bifidobacterium* associated with improved metabolic parameters. The effects of GML extended to poultry, where 0.9 g/kg supplementation reduced intestinal inflammation markers (IL-6, IL-1) in broilers [50]. Comparative studies showed that GML outperforms GTL in increasing *Bifidobacterium* and reducing *Desulfovibrio* in HFD-fed mice [42]. The addition of 0.1% lauric acid increased the abundance of *Firmicutes*, *Clostridia*, and *Bacilli* in black sea bream (*Acanthopagrus schlegelii*) [51]. Liu et al. [52] found that 0.3 g/kg of medium-chain α-monoglycerides (MG) elevated the relative abundance of genera such as *Lachnospiraceae_NK4A136_group*, along with *Proteobacteria* and *Faecalibacterium*. These microbial changes showed significant correlations with serum biochemical markers and sex hormones, suggesting a potential mechanism for improving laying performance in hens through microbiota modulation. The mechanisms involve multiple pathways: the upregulation of tight junction proteins, enhanced antioxidant capacity, and anti-inflammatory effects that collectively maintain gut barrier integrity [53]. These findings position GML as a promising dietary intervention for gut microbiota modulation, with potential applications in metabolic disease prevention and intestinal health maintenance.

#### 1.3.3. Enhancing Immunity and Combating Fatigue

The immune system is essential for protecting the body against pathogen invasion and maintaining overall health. Fatigue may occur when the immune system is actively engaged. The mechanisms involve multiple pathways: the upregulation of tight junction proteins, enhanced antioxidant capacity, and anti-inflammatory effects that collectively maintain gut barrier integrity during viral or bacterial infections. Thus, preserving optimal immune function can mitigate fatigue. The gut, recognized as the largest organ associated with the immune system, plays a crucial role in maintaining gut homeostasis and suppressing inflammation through its dynamic interactions with gut microbiota and the host immune response [54]. Studies have demonstrated that the supplementation of 0.6, 0.9, and 1.2 g/kg of GMC in broilers significantly elevated serum levels of IL-1 and IL-6 in 14-day-old chicks, potentially enhancing the intestinal immune barrier function. Additionally, GMC could modulate serum and jejunal pro-inflammatory factors, appropriately increasing cytokine levels under physiological conditions, which might confer immune-enhancing and -promoting effects [49]. MCTs enhanced the functionality of immune cells, modulated immune responses, and prevented excessive immune reactions and inflammation in pigs. Additionally, MCTs improved the antioxidant capacity and bolstered the activity of antioxidant enzymes, thereby reducing free radical damage to immune cells [53]. Medium-chain triglycerides containing C6 caproic acid and C8 octanoic acid (MCT6/8) could enhance the levels of serum immunoglobulin A (IgA) and increase the number of IgA-positive plasma cells and cup cells in the small intestine of piglets. Serum IgA, a crucial component of humoral immunity, facilitated the elimination of pathogens that penetrate the mucous membranes and strengthened the body’s defense against infections caused by pathogenic bacteria, including toxin-producing *Escherichia coli* (ETEC). IgA-positive plasma cells are the primary producers of IgA antibodies, and their increased abundance promotes the intestinal secretion of IgA, thereby bolstering the defensive function of the intestinal mucosa. Additionally, cup cells are specialized intestinal epithelial cells that secrete mucus, which protects the intestinal tract from pathogenic bacteria and fosters communication between immune cells and intestinal epithelial cells [55].

## 2. Medium-Chain Fatty Acid Triglycerides in Aquatic Animals

### 2.1. Enhanced Production Performance

The unique nutritional properties of MCTs have significant positive effects on the growth and quality of aquatic animals. Incorporating MCTs into the diet can enhance the weight gain rate (WGR), feed conversion ratio (FCR), and survival rate (SR) while simultaneously reducing organ indices such as the viscerosomatic index (VSI), hepatosomatic index (HSI), and abdominal fat rate (AFR). These effects contribute to a reduction in fat accumulation in aquatic animals. The supplementation of 1.0 g/kg of GML to the culture of large yellow croaker (*Larimichthys crocea*) proved particularly effective in increasing the body weight after 23 days of feeding. Furthermore, after 120 days of feeding, the body mass index of fish in the dosage group receiving 1.0 g/kg of GML was significantly higher than that of the control [56]. The ratio of length to caudal peduncle height was slightly greater in the GML group. This change contributed to a tendency toward a more slender body shape in the yellow croaker [57]. The addition of 0.5% SILOhealth 108Z (a mixture of short- and medium-chain 1-monoglycerides ranging from C3 to C12 with 65% 1-butyrate) to the diet of gilthead sea bream did not significantly improve the growth performance, but significantly reduced the economic feed conversion ratio (eFCR) by 4.52% (*p* < 0.05), presuming that this feed additive improved the feed efficiency by improving the gut health [58]. The addition of 0.7 g/kg and 1.05 g/kg of GML to the diet significantly enhanced the growth performance of white shrimp (*Litopenaeus vannamei*), manifested as increases in the final body weight, WGR, and specific growth rate (SGR) by 11.21~13.32%, 12.48~17.30%, and 5.52~7.59% (*p* < 0.05), respectively. Additionally, these treatments promoted lipid and protein digestion and absorption [59]. The incorporation of 2 g/kg of GML into the diet improved the growth performance of Chinese mitten crabs (*Eriocheir sinensis*), significantly increasing the WGR and SGR by 9.96% and 6.29%, respectively (*p* < 0.05), and significantly decreasing the FCR by 17.26% (*p* < 0.05), according to Fu et al. [60]. Furthermore, both medium-chain triglycerides TC6 and TC8 significantly improved the survival and growth rates of common carp (*Cyprinus carpio* L.) larvae. However, during the later stages of the experiment, the growth rate of TC8-fed carp fry began to decline, and their survival rate was lower than that of TC6-fed carp fry. This decline may be attributed to the elevated levels of ketone bodies produced during the metabolism of TC8, which could lead to metabolic disorders [61]. Lastly, the addition of 2 g/kg of GML to the feed reduced the organ coefficients of Asian seabass (*Lates calcarifer*), including the viscerosomatic index(VSI), hepatosomatic index(HSI)and intraperitoneal fat ratio(IPF), thereby effectively decreasing fat accumulation in the fish while improving the feed conversion rate, which may promote growth [62]. The improvement effect of MCTs on the production performance of aquatic animals is shown in Table 2.

It is not difficult to see that MCTs exhibit significant dose-threshold effects on the growth regulation of aquatic animals. Positive cases show that 1.0 g/kg of GML can significantly improve the body weight index and the ratio of body length to caudal peduncle height in large yellow croaker, promoting body shape optimization [56,57]. Additionally, 2 g/kg of GML significantly enhances the WGR and SGR in Chinese mitten crabs [60]. In contrast, common carp larvae fed with TC8 showed an improved initial growth rate, but subsequent growth stagnation and lower survival rate compared to the TC6 group due to ketone body accumulation [61]. This discrepancy highlights the critical influence of MCTs’ molecular forms and carbon chain lengths. Medium-chain triglycerides (e.g., TC8) have faster β-oxidation rates, prone to excessive ketone body production and metabolic disorders, while monoglycerides (e.g., GML) directly enter the liver via the portal vein for energy metabolism, significantly reducing the toxic accumulation risks. Metabolic responses to MCTs differ fundamentally among aquatic animal groups. White shrimp (Litopenaeus vannamei) shows significant growth responses to 0.7–1.05 g/kg of GML, manifested by enhanced digestive enzyme activities [59], whereas gilthead sea bream exhibits no significant growth promotion but improved feed conversion ratio when fed with 0.5% SILO-health 108Z [58]. In-depth analyses are required to clarify the underlying mechanisms.

### 2.2. Improved Flesh Quality

Parameters such as hardness, springiness, chewiness, and adhesiveness are commonly employed to describe the textural properties of fish. The textural characteristics of fish are closely related to the diameter and density of muscle fibers. Typically, muscle fiber density has a positive correlation with muscle hardness, where higher muscle fiber density is associated with greater muscle hardness [63]. Intrinsic attributes, including myogenic fiber structure, water distribution, and protein composition in muscle tissue, significantly influence the texture of fish [64]. The addition of 0.5 g/kg and 1.0 g/kg of GML enhanced the muscle texture, color, flavor, and nutritional value of greater amberjack. Specifically, it increased the muscle firmness and chewiness while improving the overall muscle quality. Furthermore, GML supplementation elevated the muscle content of free amino acids, palatable amino acids, mono-unsaturated fatty acids, poly-unsaturated fatty acids, eicosapentaenoic acid (EPA), and docosahexaenoic acid (DHA), while decreasing the ratio of saturated fatty acids to unsaturated fatty acids (SFA/UFA) [56]. Additionally, 1.0 g/kg of GML supplementation maintained the muscle quality of yellow croaker during the fasting phase, resulting in an improved body shape and color [56]. GML could enhance the muscle quality of yellow croaker through several mechanisms, which increases the content of sweet and bitter amino acids, thereby enhancing the intensity and persistence of taste, and elevates the ratio of essential amino acids (e.g., lysine) to non-essential amino acids, thereby improving the nutritional value of fish flesh. Additionally, GML boosted the content of n-3 polyunsaturated fatty acids, such as EPA and DHA, and altered the composition of volatile flavor compounds in the muscle [57]. It also increased the levels of aromatic substances, including n-hexanal, 1-pentenal, and 2-octenal, while reducing the content of 2,7-octadien-1-ol and 2-methylpentane. Consequently, GML improves muscle cohesion, modifies muscle fiber characteristics, and influences muscle metabolism to enhance texture. Moreover, GML regulates the expression of muscle growth-related genes, upregulating the levels of myogenic regulators such as *MyoD*, *Myf5*, and *MyoG*, which might promote muscle fiber proliferation. It also increased the expression of protein synthesis-related genes, such as insulin-like growth factor-1 (IGF-1) and the mechanistic target of rapamycin (mTOR), thereby facilitating muscle protein synthesis and development. The upregulation of IGF-1 and mTOR might further enhance muscle protein synthesis [57]. The addition of GML in feed improves the meat quality of yellow croaker by increasing the crude lipid content of muscle, significantly upregulating the metabolic pathways related to amino acid biosynthesis, and enhancing both the essential and non-essential amino acid content. Furthermore, it lowered the content of saturated fatty acids while increasing the ratio of unsaturated fatty acids, resulting in healthier muscle tissue. GML also activated protein digestion and the absorption pathways and signaling pathways related to muscle growth. It upregulated the expression of MyoD and Mrf5 while downregulating myostatin (MSTN), which contributed to a reduction in muscle fiber diameter, resulting in more delicate muscle tissues. Additionally, GML decreased the bitter and salty tastes of the muscle while improving the overall flavor of the meat [65]. The addition of 0.75 g/kg of GML increased the crude fat content, the thickness of the foregut muscle layer, and the water content in the back muscles of blood parrot fish (*Cichlasoma synspilum* ♀ × *Cichlasoma citrinellum* ♂), which enhanced the muscle tissue morphology and subsequently improved the muscle texture. Therefore, the inclusion of MCTs in the diet of aquatic animals effectively improved the muscle and meat quality by regulating the genes related to muscle growth and increasing the levels of amino acids and volatile flavor compounds [66]. Table 2 shows the role of MCTs in improving the muscle quality of aquatic animals.

GML exerts remarkable dose- and species-dependent effects on improving the fish meat quality. In the large yellow croaker, 1.0 g/kg of GML reduces the muscle fiber diameter and enhances the muscle toughness, while maintaining adipose metabolic homeostasis during fasting to prevent meat quality deterioration [56]. By contrast, in the blood parrot fish, 0.75 g/kg of GML increases the water content in dorsal muscles and thickness of the foregut muscle layer, but fails to significantly elevate umami amino acids (e.g., glutamic acid) or regulate volatile aldehydes as potently as in the large yellow croaker [66]. This discrepancy may be attributed to two key factors. First, species-specific differences in muscle metabolic characteristics play a role: marine fish such as the large yellow croaker have higher muscle mitochondrial density, enabling GML-induced fatty acid β-oxidation to more efficiently generate flavor precursors. In contrast, the glycolysis-dominated metabolic pattern of the blood parrot fish (*Cichlidae*) may attenuate GML’s regulatory effect on lipid oxidation pathways. Second, a dose-threshold effect is at play: the 0.75 g/kg dosage used in the blood parrot fish experiment may be below the optimal induction concentration for flavor substance synthesis (e.g., the effective dose for the large yellow croaker is 1.0 g/kg), resulting in less pronounced flavor improvement.

### 2.3. Regulated Lipid Metabolism

The liver serves as the central organ for lipid metabolism in fish, responsible for various metabolic activities, including fat synthesis, catabolism, and transport. The biological activities of fatty acid synthase (FAS), acetyl-CoA carboxylase (ACC), and lipoprotein lipase (LPL) are useful indicators of the lipid metabolism levels within the organism [67]. FAS and ACC are critical enzymes involved in fatty acid synthesis and metabolism in the liver. ACC catalyzes the conversion of acetyl-CoA to malonyl-CoA, while FAS facilitates the synthesis of fatty acids from acetyl-CoA and malonyl-CoA. The activity levels of these two enzymes can be employed to gauge the rate of fatty acid synthesis in the organism [68]. The specific details regarding the regulatory effect of MCTs on lipid metabolism in aquatic animals are shown in Table 2. White shrimp fed 0.7 g/kg and 1.05 g/kg GML showed significant increases in lipase and protease activities in the intestine, which facilitated the digestion and absorption of lipids and proteins [59]. Feeding tricaprylin (TC8) resulted in increased levels of 8:0 and 10:0 fatty acids in the neutral lipids of common carp larvae, suggesting that TC8 is partially converted to these fatty acids rather than being directly oxidized for energy, which may impair fatty acid accumulation [69]. In contrast, tricaproin (TC6) did not promote the formation of medium-chain fatty acids. Additionally, TC8 exhibited a greater ketogenic potential than TC6, indicating that it is more readily converted into ketone bodies rather than being oxidized directly for energy. This suggested that TC8’s partial conversion to 8:0 and 10:0 fatty acids contributes to fatty acid accumulation and potential damage, whereas TC6 does not induce medium-chain fatty acids. Consequently, in the later stages of the experiment, the growth rate of the TC8 group decreased, and the survival rate was reduced [61]. Studies have shown that feeding tricaprylin (TC8) can reduce the growth performance of juvenile red drum (*Sciaenops ocellatus*) by increasing blood β-hydroxybutyric acid (β-HBA) levels, which in turn elevates ketone body levels. This is consistent with the results of this study [70]. LPL serves as a key rate-limiting enzyme in lipid metabolism, converting triglycerides from chylomicrons and very low-density lipoproteins (VLDL) into free fatty acids and glycerol, thereby effectively reducing the total cholesterol (TC) levels in the blood [71]. The addition of 2 g/kg of GML significantly increased the activity of LPL in the liver of common goldfish (*Carassius auratus*), promoting lipolysis and oxidation while regulating the TG balance. This intervention reduced the levels of TG, TC, and LDL-C in serum, simultaneously increasing HDL-C and improving lipid metabolism. Additionally, GML enhanced the activity of amylase, LPL, and protease in the intestines of common goldfish, facilitating nutrient and energy absorption from feed. The improved activities of amylase, LPL, and protease in the intestinal tract further supported nutrient absorption [66]. GML reduced lipid deposition in Asian seabass (*Lateolabrax maculatus*) by decreasing the abdominal fat index, as well as the activities of FAS and ACC, while also diminishing liver fat deposition. Moreover, GML could enhance LPL activity and promote lipid oxidation to facilitate lipolysis, consequently lowering serum levels of TG, TC, and LDL-C while increasing HDL-C levels [72]. Additionally, the incorporation of 1.5 g/kg of GML reduced HFD-induced excessive lipid accumulation and damage in zebrafish (*Danio rerio*) liver. HFD led to significant obesity in zebrafish, with increased hepatic total TC and triglyceride TG levels, causing severe lipid deposition in the liver and a severe imbalance of the body’s antioxidant system [73]. GML decreased lipid synthesis in zebrafish liver and promoted liver lipolysis, thereby mitigating HFD-induced lipid accumulation. Additionally, GML enhanced the hepatic antioxidant capacity, upregulated the gene expression of anti-inflammatory factors TGF-β1 and IL-10, and downregulated pro-inflammatory cytokines (such as tumor necrosis factor-α (TNF-α), interleukin-1β (IL-1β), and interleukin-6 (IL-6)), thereby alleviating HFD-induced oxidative stress and inflammatory responses. GML also significantly enhanced the expression of AMP-activated protein kinase (AMPK), which is inhibited by HFD [74]. The addition of 1 g/kg of GML to the feed promoted LPL activity, reduced the lipid content, enhanced the expression of genes related to TG and fatty acid catabolism, and decreased plasma TG levels in white shrimp [75]. Moreover, the inclusion of 1.5 g/kg GML improved lipid metabolism and protects the liver from damage in cage-farmed pompano (*Trachinotus ovatus*) juveniles. GML sig-nificantly reduced TG levels, while TC content was diminished in the 1.5 g/kg, 2 g/kg, 2.5 g/kg groups. Concurrently, HDL levels were significantly increased, and LDL levels were decreased in the 0.5 g/kg and 1 g/kg GML groups, with a notable reduction in the synthesis and expression of fatty acids in the 1.5 g/kg, 2 g/kg, 2.5 g/kg GML groups [76].

The regulation of hepatic lipid metabolism by GML in fish exhibits a significant dose-threshold effect: in common goldfish, 2 g/kg of GML promoted lipid decomposition by activating LPL, reducing serum TG and TC, while increasing HDL-C levels [62]; in zebrafish, 1.5 g/kg GML alleviated hepatic lipid deposition induced by HFD, but its inhibitory effect on FAS was only significant in the HFD model, with weak effects in the normal diet group [73,74]. The core contradiction lies in metabolic pathway competition: high-dose GML (≥1.5 g/kg) inhibits FAS activity via the AMPK pathway, whereas low-dose (0.5–1.0 g/kg) GML may preferentially participate in energy metabolism rather than lipid regulation, leading to effect differences. Additionally, as a model organism, the zebrafish liver shows lower sensitivity to GML-mediated lipid metabolism compared to economically farmed fish like common goldfish. The HFD model amplifies the intervention effect of GML by artificially inducing lipid metabolic disorders. The carbon chain length also influences fat metabolism: the β-oxidation rate of C8 fatty acid (TC8) is faster than that of C6 (TC6), prone to generating excessive ketone bodies and causing metabolic toxicity, while GML (C12 monoglyceride) undergoes milder metabolism with a lower toxicity risk.

### 2.4. Boosted Immunological Activity

Superoxide dismutase (SOD) and catalase (CAT) are interrelated antioxidant enzymes that play crucial roles in the body by scavenging harmful superoxide radicals (O_2_) produced within the organism, thereby preventing cellular damage. Their activity directly influences the level of bioimmunity [77]. Toll-like receptors (TLRs) are a class of pattern recognition receptors (PRRs) capable of recognizing pathogen-associated molecular patterns and damage-associated molecular patterns, thus initiating innate immune responses [78]. Furthermore, the addition of MCTs positively affects immune activity. The addition of 0.35 g/kg of GML enhanced the non-specific immunity of white shrimp by activating immune signaling pathways, such as the Toll signaling pathway and the immunodeficiency (Imd) signaling pathway. These pathways promote the expression of antimicrobial peptides (AMPs) and enhanced the antimicrobial capacity of white shrimp. Furthermore, GML might stimulate the expression of antioxidant enzymes, such as SOD and CAT, which scavenge free radicals and alleviate oxidative stress, thereby enhancing the immune defense of white shrimp [59]. An addition of 2.0 g/kg of GML could upregulate the expression of alpha-fetoprotein (ALF) and lysozyme (LZM) while downregulating the expression of caspase-3, indicating that GML enhances the immunity of Qihe crucian carp (*Carassius auratus*) by regulating immune-related genes. Simultaneously, GML supplementation could upregulate the expression of Toll-like Receptor 1 (TLR1) and Toll-like Receptor 2 (TLR2), key proteins in the Toll signaling pathway that are involved in regulating the innate immune response [79]. Unlike antibiotics that function by inhibiting bacterial cell wall synthesis [80], MCTs (such as GML) enhance innate immunity by activating the TLR signaling pathway. However, their effects on specific immunity (e.g., antibody production) have not been compared with traditional immunostimulants (such as β-glucans) in terms of dose–response relationships. Further validation is needed to assess their sustainability in long-term immune protection. Consequently, the increased expression of TLR1 and TLR2 would activate the Toll signaling pathway, thus enhancing the immunity of Chinese mitten crab [60]. Different from the addition of 0.35 g/kg in white shrimp [59], when 1.8 g/kg of GML was added to the diet of hybrid grouper, the serum immunoenzyme activities initially increased and then decreased [81], suggesting that high doses may trigger metabolic overload or feedback inhibition. The expressions of TNF-α and IL-6 in head kidney tissue were significantly inhibited, while the expression of TLR22 was significantly increased [81]. Additionally, 1.0 g/kg of GML could enhance the humoral immunity of *L. vannamei* by activating Toll-like receptors and the immune deficiency pathway, thereby improving the phagocytic and antibacterial abilities of shrimp blood cells [75]. The enhancement of immune activity in aquatic animals by MCTs is shown in Table 2

### 2.5. Modulated Intestinal Flora

SILOhealth 108Z promotes the growth of *Lactobacillus* and inhibits the growth of γ-proteobacteria in the intestine of gilthead seabream by releasing organic acids and lowering the intestinal pH. Additionally, monoglycerides exhibit an amphiphilic structure that interacts with bacterial cell membranes, disrupting membrane integrity and leading to bacterial death [58]. As shown in Table 2, adding different doses of MCTs can regulate the intestinal flora of aquatic animals. The administration of 1.0 g/kg and 2.0 g/kg of GML to Chinese mitten crabs supports the growth of beneficial bacteria. In the 2.0 g/kg GML group, the abundance of the gut microbiome played a critical role in regulating host health by influencing metabolism and disease development [82]. It had an irreplaceable impact on preventing infectious diseases and regulating the digestion and metabolism of carbohydrates and lipids, while also maintaining host immune homeostasis [83]. A rich diversity of microbial flora enhanced intestinal microecological stability, increased resistance to foreign bacteria and inflammation, and reduced susceptibility to these threats [84]. The microbial community in the intestinal tract of aquatic animals was diverse, comprising various microbial groups, including archaea, bacteria, protozoa, fungi, and viruses. Numerous studies have demonstrated that the addition of MCTs could improve the composition and abundance of intestinal flora in aquatic animals, thereby enhancing their microbial community structure and reducing inflammatory responses. GML increased the intestinal flora of black sea bream, significantly enhancing the relative abundance of the phylum *Firmicutes*, as well as the genera *β-amoeba*, *γ-amoeba*, and *Clostridium*, according to the study by Sami et al. [51]. The thick-walled phylum increased, and the Gram-negative bacterium *Shewanella*, isolated for the first time from the intestinal contents of the GML group, significantly increased. *Shewanella* may contribute to improved intestinal health and immunity while inhibiting the growth of harmful bacteria and enhancing the intestinal environment by reducing inflammation. The study by Sami et al. [51] noted these effects, including the inhibition of harmful bacteria and the improvement of the inflammatory response. GML significantly increased the Shannon and PD_whole_tree indices of the intestinal flora in zebrafish (*Danio rerio*), enhancing microbial diversity and promoting the growth of beneficial bacteria such as *Clostridium* spp., *Hibiscus* spp., and *Vibrio* spp., thereby creating a favorable environment for nutrient absorption [73]. The addition of 0.75 g/kg of GML markedly improved the α-diversity of the intestinal microbiota and upregulated the abundance of *Cetobacterium*, known for its ability to produce vitamin B12 and butyric acid. Furthermore, GML downregulates the abundance of some non-dominant bacteria, such as *Pseudonocardia*, *Carnobacterium*, and *Staphylococcus*, contributing to the regulation of intestinal health and energy metabolism. It also increases the abundance of enzyme-producing bacteria, including *Shewanella* and *Vibrio*, which secrete various digestive enzymes, aiding blood parrot fish in better digesting food and absorbing nutrients. Additionally, the study found that adding 0.75 g/kg of GML enhances the activity of midgut lipase, intestinal protease, and posterior intestinal lipase, improving digestion in grass carp. The α-diversity of intestinal flora increased, and the abundance of *Clostridium*, *Vibrio*, and *Shewanaceae* was upregulated, facilitating the colonization and growth of *Clostridium* and zymogenic bacteria. Conversely, the abundance of Firmicutes, *Nocardia*, *Carnobacterium*, and *Staphylococcus* was downregulated, demonstrating the bacteriostatic effects of GML [66]. Following a challenge test with *Vibrio parahaemolyticus*, the activities of acid phosphatase (ACP) and alkaline phosphatase (AKP) in the hybrid grouper groups receiving 1.8 g/kg and 2.4 g/kg of GML increased; moreover, the mortality rate was lower than that of the control group. Additionally, the relative abundance of *Firmicutes* and *Bacillus* increased, highlighting GML’s significant regulatory effect on the abundance of intestinal flora. The addition of 2.4 g/kg of GML improved the Shannon, Chao1, and ACE indices, thereby enhancing the α-diversity of intestinal flora in this group [81]. GML also increased the abundance of beneficial bacteria in *Litopenaeus vannamei*; however, the administration of 1.5 g/kg of GML adversely affected the stability of the intestinal microbiome by significantly upregulating the levels of intestinal antimicrobial peptide-related genes and tumor necrosis factor-α [75]. Moreover, the addition of 1.8 g/kg of GML alleviated oxidative stress and restored the balance of intestinal flora disrupted by variations in salinity in juvenile hybrid *Epinephelus*. GML supplementation delayed disturbances in the intestinal flora, inhibited the colonization of harmful bacteria such as *Vibrio*, and promoted the abundance of beneficial bacteria like *Bacillus* [85].

In-depth analysis shows that crustaceans (such as shrimp) have lower intestinal flora diversity, and the antibacterial effect of MCTs can directly improve intestinal health, while fish (such as gilthead seabream) have more complex intestinal microecology, and the effect of short-chain fatty acid monoesters is easily offset by bacterial metabolism, requiring higher doses or longer action times to show effects. This difference is essentially caused by inherent differences in metabolic enzyme activities between species (such as the activity of liver ketone body metabolic enzymes in carp being lower than that in perciform fish) and intestinal flora structure (such as the abundance of Vibrio in shrimp intestines being higher than that in fish). In addition, the regulation of aquatic animal flora diversity by GML also has a dose-dependent effect. Low doses of 0.75–1.0 g/kg of GML promote the proliferation of symbiotic bacteria such as *Shewanella* by destroying the cell membranes of pathogenic bacteria (such as *Vibrio*) and releasing ecological niches [73]; high doses of 1.5–2.4 g/kg of GML may excessively damage membranes, simultaneously inhibiting beneficial bacteria and leading to flora structure imbalance [75,81]. In conclusion, dietary supplementation of MCTs can improve the intestinal flora of aquatic animals through several mechanisms: (1) increasing the abundance of *Firmicutes* and the diversity of intestinal flora, (2) promoting the growth of beneficial bacteria, and (3) inhibiting the growth of harmful bacteria.

**Table 2:** Effects of MCT additives on aquatic animals.

MCTs Optimal Additive	Test Object	Result	Reference
1.0 g/kg of GML	Large yellow croaker (*Larimichthys crocea*)	Weight, body mass index ↑, muscle texture, color, flavor and nutritional value ↑	[56]
0.75 g/kg of GML	Greater amberjack (*Seriola dumerili*)	Height ↑, body length ↑, FAA ↑, EPA ↑, DHA ↑, cohesion ↑ 2,7-octanediene-1-ol ↓, 2-methylpentane ↓ MRFs ↑ (*MyoD* ↑, *Myf-5* ↑, *MyoG* ↑) IGF-1 ↑, mTOR ↑	[57]
5 g/kg of SILOhealth 108Z	Gilthead sea bream (*Sparus aurata*)	eFCR ↓, intestinal tract pH ↓, *Lactobacillus* ↑, γ- proteobacteria ↓	[58]
0.7 g/kg of GML	White shrimp (*Litopenaeus vannamei*)	Weight ↑, WGR ↑, SGR ↑, lipase ↑, protease ↑, *Toll* ↑, *Imd* ↑, AMPs ↑	[59]
2 g/kg of GML	Chinese mitten crabs (*Eriocheir sinensis*)	ALF, LZM expression level ↑, caspase-3 expression level ↓, Bacillota, *Shewanella* abundance ↑	[60]
TC6 TC8	Common carp (*Cyprinus carpio* L.) larvae	Survival ↑, growth ↑, liver size ↑, hepatocyte volume ↑, In TC8 group: lipids 8:0 and 10:0 levels ↑	[61]
2 g/kg of GML	Asian seabass (*Lates calcarifer*)	VSI, HSI, IPF ↓; AMY, LPL ↑; TG, TC, LDL ↓; HDL ↑	[62]
0.75 g/kg of GML	Blood parrot fish (*Cichlasoma synspilum* ♀ × *Cichlasoma citrinellum* ♂)	Muscle fat ↑, gut thickness ↑, water content ↑ α-diversity ↑, *Cetobacterium* ↑, *Shewanella* ↑, *Vibrio* ↑, *Pseudonocardia* ↓, *Carnobacterium* ↓, *Staphylococcus* ↓	[66]
2 g/kg of GML	Asian seabass (*Lateolabrax maculatus*)	Abdominal value ratio, FAS and ACC activity ↓, LPL activity ↑	[72]
1.5 g/kg of GML	Zebrafish (*Danio rerio*)	Lipogenesis ↓, lipolysis ↑, anti-inflammatory ↑, proinflammatory ↓, diversity ↑	[73]
2.14299 g/kg of GML	White shrimp (*Penaeus vannamei*)	Antibody ↑, phagocytosis ↑, antibacterial ↑, probiotics ↑, AMP genes ↓, TNF-α ↓, LPS ↓, TG ↓	[75]
1.5 g/kg of GML	Cage-farmed pompano (*Trachinotus ovatus*)	TG, TG, LDL ↓, HDL ↑	[76]
1.7 g/kg of GML	Hybrid grouper (*Epinephelus fuscoguttatus* ♀ × *E. lanceolatus* ♂)	ACP, AKP, and LZM activity ↓, TLR22 expression ↑ *Firmicutes* and *Bacillus* ↑, Sob, Chao1, and ACE index ↑	[81]
1.8 g/kg of GML	Juvenile grouper (*Epinephelus* spp.)	Oxidative stress ↓, gut flora imbalance ↓, *Vibrio* ↓, *Bacillus* ↑	[85]

Note: In the table, “↑” indicates an increase in the content of the substance, and “↓” indicates a decrease in the content of the substance. GML denotes glycerol monolaurate, FAA denotes free fatty acid, EPA denotes eicosapentaenoic acid, DHA denotes docosahexaenoic acid, MRFs denotes muscle regulatory factors, IGF-1 denotes insulin-like growth factor-1, mTOR denotes mammalian target of rapamycin, SILOhealth 108Z denotes a mixture of short- and medium-chain 1-monoglycerides ranging from C3 to C12 with 65% 1-butyrate, eFCR for economic feed conversion ratio, WGR denotes weight gain rate, SGR denotes specific growth rate, AMPs denotes antimicrobial peptides, ALF denotes anti-lipopolysaccharide factor, LZM denotes lysozyme, TC6 denotes tricaproin, TC8 denotes tricaprylin, VBR denotes viscerosomatic index, LBR denotes hepatosomatic index, AFP denotes abdominal fat percentage, AMY denotes amylase, LPL denotes lipoprotein lipase, TG denotes triglyceride, TC denotes total cholesterol, LDL denotes low-density lipoprotein cholesterol, HDL denotes high-density lipoprotein cholesterol, FAS denotes fatty acid synthase, ACC denotes acetyl-CoA carboxylase, TNF-α denotes tumor necrosis factor-alpha, LPS denotes lipopolysaccharide, ACP denotes acid phosphatase, AKP denotes alkaline phosphatase, and TLR22 denotes Toll-like receptor 22.

## 3. Conclusions

MCTs demonstrate significant potential as functional feed additives in aquaculture systems. Scientific evidence indicates that MCT supplementation can achieve the following: (1) effectively reduce the disease incidence and enhance survival rates in cultured aquatic species; (2) improve the growth performance and production efficiency through enhanced nutrient utilization; and (3) optimize the product quality by increasing the muscle protein content and improving sensory characteristics. Furthermore, MCTs exhibit immunomodulatory properties that strengthen disease resistance while maintaining overall animal health. Their ability to modulate the gut microbiota composition, promoting beneficial bacterial populations while suppressing pathogenic species, contributes to improved intestinal homeostasis. These multifaceted benefits, including demonstrated antibacterial, antiviral, anti-inflammatory, and metabolic regulatory activities, position MCTs as a superior alternative to traditional feed additives in sustainable aquaculture. Unlike antibiotics with the risk of microbial resistance or synthetic chemicals with residue concerns, MCTs provide a biodegradable and non-toxic mechanism, capable of simultaneously enhancing disease resistance, nutrient utilization, and intestinal health.

Despite the significant application potential of MCTs in aquaculture, current research harbors several limitations that concurrently illuminate future research directions. In terms of research scope, the existing studies predominantly focus on specific species, leaving the metabolic response heterogeneity of Cyprinidae, Sparidae, and other taxa unaddressed. Such gaps in species-specific toxicity and metabolic mechanisms necessitate cross-species comparative studies to establish dose-effect models based on key enzyme expression (e.g., AMPK, PPARα). Concerning the research duration and ecological impacts, short-term experiments (30–120 days) fail to cover chronic toxicity assessments across the entire culture cycle, and systematic data on the cascade effects of MCT water residues on plankton communities remain lacking, underscoring critical voids in long-term ecological safety research. For dose–response relationships, the broad dosage gradient fails to capture species-specific optimal thresholds, requiring proteomics-integrated dose-ramp experiments to identify physiological inflection points and overcome precision bottlenecks. Additionally, research on environmental factor interactions remains insufficient: the regulatory mechanisms of ecological parameters (e.g., water temperature, salinity) on MCT metabolic pathways require clarification, and constructing a three-dimensional “dosage–environment–physiology” regulation model will provide pivotal theoretical support for industrial applications. Additionally, from a commercial perspective, the application of MCTs needs to break through the cost–benefit bottleneck. Currently, the production cost of GML is approximately 3–5 times that of antibiotics (calculated based on adding 1.0 g/kg of GML to each ton of feed). However, whether the increased weight gain rate in aquatic products can cover the cost increment requires input–output modeling combined with different aquaculture modes. Building on these limitations, future MCT research in aquatic animals will follow a logical framework: first, prioritize species-specific optimal dosages tailored to different taxa and farming environments; concurrently, explore action mechanisms to underpin the development of novel feed additives; additionally, assess safety to ensure secure aquaculture applications; and ultimately, expand MCT applications to diverse farmed species and develop MCT-based products to advance sustainable aquaculture.

## Figures and Tables

**Table 1:** Bacteriostatic effect of MCT additives.

MCT Additives	Test Object	Result	Reference
0.4–3.6 mmol/L GML	*Bacillus stearothermophilus*	Concentration ↑, spores’ heat resistance ↓, GML inactivation rate ↑	[21]
0.1% GML microemulsion	Mooncake	Anti-mildew fresh-keeping effect ↑	[22]
0.035% GML compound	Sausage	Colonies number ↓, anti-corrosion effect ↑	[10]
5% GML gel	*Bacillus anthracis*/ *Bacillus subtilis*	Anthrax spores contamination ↓, *B. subtilis* eradication ↑	[23]
TGML	Food-borne pathogens	MIC, MBC ↓ Membrane permeability ↑	[24]
Monoglyceride-LNCs	*Staphylococcus aureus*, horse erythrocytes	Bactericidal effect, antibacterial activity ↑	[26]
8 mg/disc GML	Infant formula milk powder	Potent antibacterial	[27]

Note: In the table, “↑” indicates an increase in the content of the substance, and “↓” indicates a decrease in the content of the substance. GML denotes glycerol monolaurate, TGML denotes glycerol monolaurate, MIC denotes minimum inhibitory concentration, MBC minimum bactericidal concentration, LNCs denotes lipid nanocapsules.

## Data Availability

For this review article, no new data were generated. The data analyzed in this study were retrieved from publicly available scientific literature sources. All the relevant research papers, reports, and datasets used in the compilation of this review can be accessed through major academic databases such as Web of Science, PubMed, and Scopus. The specific references for each data point or finding are clearly cited within the manuscript, enabling readers to trace them back to the original sources. In cases where data from specific research institutions or repositories were utilized, the corresponding URLs or access details are provided in the reference list. Should readers require further clarification or additional data related to the topics covered in this review, they are encouraged to contact the corresponding author. The corresponding author will make every effort to assist, subject to the terms and conditions of the original data providers.
